# Impact of Transvalvular Cardiac Implantable Electronic Devices on Outcomes Following Tricuspid Transcatheter Edge-to-Edge Repair

**DOI:** 10.1016/j.shj.2025.100744

**Published:** 2025-10-24

**Authors:** Salman Zahid, Muhammad Ahmad, Saket Sanghai, Charles A. Henrikson, Scott Chadderdon, Firas Zahr

**Affiliations:** a Division of Cardiovascular Medicine, Knight Cardiovascular Institute, Oregon Health & Science University, Portland, Oregon, USA; b Department of Medicine, Khyber Medical College, Peshawar, Pakistan

**Keywords:** Cardiac implantable electronic device, CIED, Transcatheter tricuspid repair, Structural heart intervention, Tricuspid valve, Transvalvular lead

## Abstract

• Cardiac implantable electronic devices (CIEDs) may contribute to or exacerbate TR, but their impact on tricuspid transcatheter edge-to-edge repair is unclear.
• Presence of CIEDs was not associated with higher mortality or adverse events.
• Tricuspid transcatheter edge-to-edge repair appears safe and effective in patients with transvalvular CIED leads.

Individuals with tricuspid regurgitation (TR) commonly have cardiac implantable electronic devices (CIEDs), which may exacerbate TR through leaflet impingement and right-sided chamber remodeling.^1^ Surgical treatment of TR carries high morbidity and mortality, with CIED presence implicated as an independent predictor of recurrent TR and mortality.^2^ However, the impact of CIED presence on outcomes following transcatheter tricuspid edge-to-edge repair (T-TEER) remains understudied. We aimed to assess outcomes in patients undergoing T-TEER with and without transvalvular CIEDs, using a large, multicenter real-world data set.

A retrospective analysis was conducted using the TriNetX Global Research Network, which provides access to deidentified electronic health records from over 120 health care organizations, mostly in the United States. As per Health Insurance Portability and Accountability Act §164.514(a), only deidentified data were used; hence, ethical approval was not applicable.

Individuals ≥18 years of age who underwent T-TEER between January 1, 2015, and December 31, 2024, were identified using the International Classification of Diseases (ICD)-10-Procedure Coding System code 02UJ3JZ. Patients were divided into 2 cohorts based on CIED status (ICD-10: Z95.0, Z45.0 and Z95.810) before T-TEER, whereas those with leadless CIED (ICD-10-Procedure Coding System: 02HK3NZ and CPT: 33274) were excluded. (Fig. 1). Baseline characteristics including demographic variables (age, sex, race, and ethnicity), comorbidities (ischemic heart disease, hypertension, chronic kidney disease, thyroid disease, and liver disease), and concurrent medication (diuretics, beta blockers, angiotensin-converting enzyme inhibitors, angiotensin receptor blockers) use were compared between the 2 cohorts.

**Figure 1:**
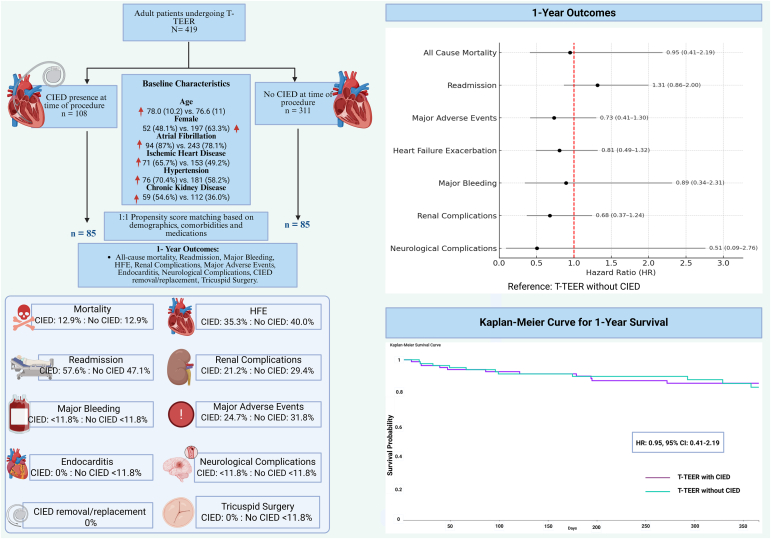
Impact of transvalvular cardiac implantable electronic devices (CIEDs) on outcomes following T-TEER. This figure illustrates the patient population and main findings. Propensity score matching was performed to create 2 cohorts of patients undergoing T-TEER, those who had a concomitant CIED and those without CIED. Primary and secondary outcomes were similar across both cohorts, with no significant differences observed. Outcomes are reported as HR with 95% CI. As per TriNetX policy, outcomes with <10 events are reported as <11.8% (<10) to maintain patient confidentiality. HFE was defined as occurence of acute pulmonary edema or use of intravenous diuretics; major adverse events were defined as the composite of death, endocarditis, acute kidney failure, and tricuspid valve surgery; major bleeding was defined as the need for central and peripheral blood transfusion; renal complication was defined as the composite of acute kidney failure and need for dialysis; neurological complication was defined as the composite of cerebral infarction, subarachnoid, intracerebral and intracranial hemorrhage, or transient ischemic attack. Abbreviations: CIED = cardiac implantable electronic device, HFE = heart failure exacerbation; HR = hazard ratio; T-TEER = tricuspid transcatheter edge-to-edge repair.

To minimize baseline confounding, 1:1 propensity score matching (PSM) was performed using greedy nearest-neighbor matching with a caliper of 0.1 pooled standard deviations. Matching was performed using baseline demographic and clinical variables.

Outcomes including all-cause mortality, readmissions, heart failure exacerbation (HFE), renal complications, major bleeding, major adverse events, neurological complications, endocarditis, CIED revision/removal, and tricuspid reintervention were compared between the 2 cohorts at 30-day and 1-year follow-ups (Fig. 1). Cox proportional hazards models were used to calculate hazard ratios (HRs) along with their 95% CI, and the log-rank test was applied to compare survival distribution between the 2 cohorts. *p* < 0.05 was considered significant. As per TriNetX policy, outcomes with 10 or fewer events were reported as <10 or <11.8% (in the PSM cohort) to maintain patient confidentiality.

Among 419 patients undergoing T-TEER, 108 had CIED, whereas 311 did not (Fig. 1). Before matching, CIED patients were older, more commonly males, and had a significantly higher prevalence of atrial fibrillation (87.0 vs. 78.1%, *p* = 0.045), heart failure (94.4 vs. 79.4%, *p* < 0.001), hypertension (70.4 vs. 58.2%, *p* = 0.025), diabetes mellitus (33.3 vs. 20.3%, *p* = 0.006), thyroid disorders (36.1 vs. 23.8%, *p* = 0.013), metabolic disorders (83.3 vs. 61.1%, *p* < 0.001), and chronic kidney disease (54.6 vs. 36.0%, *p* = 0.001), along with a higher use of diuretics (90.7 vs. 75.9%, *p* = 0.001) and beta-blockers (75.9 vs. 62.4%, *p* = 0.011). Following propensity score matching, 170 patients (85 per cohort), with well-balanced baseline profiles (standardized differences <0.1 for most covariates) were included. There were no significant differences in demographics, baseline comorbidities, medication usage, or laboratory parameters including left ventricular ejection fraction (mean: CIED: 55.1% ± 11.6% versus no CIED: 57.9% ± 7.7%, *p* = 0.368).

After PSM, there were no significant differences in short-term (30-day) outcomes. The incidence of mortality was low (<10 events, *p* = 0.65) in both cohorts. There was an increase in readmissions in the CIED cohort (42.4 vs. 28.2%; *p* = 0.054), whereas no differences could be observed in other outcomes including major adverse events, major bleeding, HFE, and renal complications (all *p* > 0.05). At 1 year, mortality was similar between patients with and without transvalvular CIEDs (CIED: 12.9% vs. no CIED: 12.9%; HR: 0.95; 95% CI: 0.41–2.19; *p* = 0.900) (Fig. 1). No significant differences were observed in readmissions (CIED: 57.6% vs. no CIED: 47.1%; HR: 1.32; 95% CI: 0.87–2.00; *p* = 0.201), major adverse events (CIED: 24.7% vs. no CIED: 31.8%; HR: 0.74; 95% CI: 0.42–1.30; *p* = 0.287), or HFE (CIED: 35.3% vs. no CIED: 40.0%; HR: 0.81; 95% CI: 0.49–1.32; *p* = 0.389). Other outcomes such as renal complications, major bleeding, and neurological complications were infrequent and did not significantly differ by CIED status. Left ventricular ejection fraction (CIED: 54.9 ± 11.31 versus no CIED: 55.60 ± 8.87, *p* = 0.690) did not differ significantly, and the incidence of new onset arrhythmias remained low in both cohorts (<10 events). There were no instances of endocarditis, surgical tricuspid reinterventions, or CIED related complications, including CIED removal or replacement in the CIED cohort.

In the unmatched analysis, incidence of readmissions were higher in the CIED cohort (CIED: 59.3% vs. No CIED: 46.6%; *p* = 0.024), while all other outcomes including all-cause mortality (CIED: 14.8% vs. No CIED: 11.9%; *p* = 0.432), major adverse events (CIED: 30.6% vs. No CIED: 28.3%; *p* = 0.655) and others (*p* > 0.05 for all) remained nonsignificant.

In this large matched retrospective analysis, we found that the presence of CIEDs did not adversely affect outcomes following T-TEER. These findings support the safety of T-TEER in this growing subgroup of patients with complex clinical characteristics.

Our findings are concordant with results from the TRILUMINATE (Trial to Evaluate Cardiovascular Outcomes in Patients Treated With the Tricuspid Valve Repair System) pivotal trial, which showed no differences in 30-day or 1-year outcomes in patients with and without CIEDs undergoing tricuspid repair with the TriClip system.^3^ Similar results were also reported by a multicenter study evaluating the impact of CIED leads on outcomes after transcatheter tricuspid valve annuloplasty.^4^ Another analysis of the Transcatheter Tricuspid Valve Repair in Spain registry also reported no differences in the primary endpoint (mortality, heart failure-related hospitalization, and tricuspid valve reintervention) and device implantation success in patients with and without CIED.^5^ Our real-world results affirm these findings in a broader, unselected patient population across diverse US health systems. The low incidence of lead revisions/removal and repeat surgical tricuspid intervention suggests that T-TEER may be safely performed in patients with CIEDs, when anatomically feasible.

Our study has several limitations. First, reliance on administrative coding may introduce misclassification bias. Second, we were unable to differentiate between active pacing, ICD, left bundle, or cardiac resynchronization and defibrillator therapy leads, and the number of implanted leads could not be ascertained. Third, echocardiographic parameters and device-related data were not available, preventing distinction between CIED-related and CIED-associated TR or assessment of the impact of T-TEER device type, lead type, or pacing modality on outcomes. Lastly, residual confounding cannot be fully excluded despite rigorous matching.

In summary, after adjusting for baseline differences, the presence of transvalvular CIED leads was not associated with increased mortality, adverse events, or procedural complications. However, the sample size was relatively small, and the findings should be interpreted as suggestive rather than definitive. Larger, prospective studies are needed to confirm these observations.

## Ethics Statement

This study used deidentified publicly available data. Ethical approval was deemed not applicable.

## Funding

The authors have no funding to report.

## Disclosure Statement

The authors report no conflict of interest.
